# Evaluation of the effect of *MTNR1B* rs10830963 gene variant on the therapeutic efficacy of nateglinide in treating type 2 diabetes among Chinese Han patients

**DOI:** 10.1186/s12920-021-01004-y

**Published:** 2021-06-12

**Authors:** Jin-Fang Song, Jie Zhang, Ming-Zhu Zhang, Jiang Ni, Tao Wang, Yi-Qing Zhao, Naveed Ullah Khan

**Affiliations:** 1 Department of Pharmacy, Affiliated Hospital of Jiangnan University , No.1000, Hefeng Road, Wuxi, 214000 China; 2 School of Pharmacy, Wannan Medical College, Wuhu, China; 3 Department of Pharmacy, Shandong Province Third Hospital, Jinan, 250000 China; 4 Department of Endocrinology, Affiliated Hospital of Xuzhou Medical College, Xuzhou, 221000 China; 5 Department of Pharmaceutics, College of Pharmaceutical Sciences, Soochow University, Suzhou, Jiangsu China

**Keywords:** *MTNR1B* rs10830963, Genetic variant, Nateglinide, Type 2 diabetes

## Abstract

Genetic polymorphisms in the *MTNR1B* gene is associated with type 2 diabetes mellitus (T2DM); however, there is no evidence about its impact on the therapeutic efficacy of nateglinide. This prospective case–control study was designed to investigate the effect of *MTNR1B* rs10830963 gene variant on the therapeutic efficacy of nateglinide in treating T2DM. We genotyped untreated T2DM patients (N = 200) and healthy controls (N = 200) using the method of the high resolution of melting curve (HRM). Newly diagnosed T2DM patients (n = 60) with CYP2C9*1 and *SLCO1B1* 521*TT* genotypes were enrolled and given oral nateglinide (360 mg/d) for 8 weeks. The outcome was measured by collecting the venous blood samples before and at the 8th week of the treatment. The risk G allelic frequency of *MTNR1B* rs10830963 was higher in T2DM patients than the healthy subjects (*P* < 0.05). Post 8-week of treatment, newly diagnosed T2DM patients showed a less reduction in fasting plasma glucose levels and less increase in the carriers of genotype CG + GG at rs10830963 when compared with the CC genotype (*P* < 0.05). *MTNR1B* rs10830963 polymorphism was associated with the therapeutic efficacy of nateglinide in T2DM patients. Also, the CC homozygotes had a better effect than G allele carriers.

*Trial registration* Chinese Clinical Trial Register ChiCTR13003536, date of registration: May 14, 2013.

**Supplementary Information:**

The online version contains supplementary material available at 10.1186/s12920-021-01004-y.

## Introduction

Type 2 diabetes mellitus (T2DM) is a chronic genetic heterogeneous disease with multiple genes and multiple environmental factors. In order to effectively control the glycemic level and to reduce the risk of secondary damage to cardiovascular, cerebrovascular, kidney and other organs, most patients with T2DM require treatment with hypoglycemic drugs [1]. Oral hypoglycemic therapy helps in achieving glycemic control in patients with type 2 diabetes. Clinical practices have found that the same drug regimen used on different patients showed significant individual differences in the efficacy of the hypoglycemic. The genetic variation of the genes involved in insulin resistance and islet beta-cell functioning which may ultimately affect the susceptibility and disease progression of T2DM. This will result in further affecting the patient's responsiveness to drug therapy.

Nateglinide is an important non-sulfonylurea oral hypoglycemic agent that improves blood glucose levels by promoting insulin secretion from pancreatic islet beta cells. However, the efficacy of the drug varies from each individual [2, 3]. The mechanism of the difference in the efficacy and adverse effects of nateglinide remains unclear. It is thought to vary based on the genetic conditions of the drug transporters, drug-metabolizing enzymes, drug receptors, and T2DM susceptibility genes [3]. There are a few types of research that attribute this difference may be due to the genetic polymorphisms of cytochrome P450 2C9 (*CYP2C9*) and genetic polymorphism in the *SLCO1B1* gene encoding organic anion transporting polypeptide 1B1 (OATP1B1) [4]. It is found that due to the genetic polymorphism of the enzymes mentioned above and their impact on the pharmacokinetic process of nateglinide might contribute to the difference in the efficacy. But, this could not elucidate the complete mechanism of action by which the same nateglinide therapy results in various therapeutic responses [5–7].

In pharmacodynamics, the onset of nateglinide is rapid but with a shorter duration of action, compared with sulfonylureas. Thus it is able to significantly reduce postprandial hyperglycemia as well as improve glycemic control in T2DM [8, 9]. In addition, other reports also showed that nateglinide had a good effect on improving insulin resistance [10–12]. Therefore, it is understood that genetic polymorphisms affecting insulin resistance or islet cell function may impact the efficacy of nateglinide treatment on T2DM patients. Hence it is important to study the effect of genetic variation on individual differences in the efficacy of nateglinide drugs for guiding clinical rational drug use and optimizing T2DM treatment strategies.

Three genome-wide association studies (GWAS) among the European populations conducted during the recent years have found that *MTNR1B* gene mutations have an association with the increased risk of T2DM, increased fasting plasma glucose (FPG), and decreased insulin secretion [13–15]. Among the observed risk variants, *MTNR1B* rs10830963(C>G) was the most strongly associated one with FPG [15]. Subsequently, it was also confirmed that *MTNR1B* rs10830963 had an association with increased FPG and increased risk of T2DM in Chinese Han population [16, 17]. Further, pharmacogenomic studies have shown that variants in genes associated with insulin secretion and insulin sensitivity, such as *KCNQ1*, *SLC30A8*, *SLC22A1*,* TCF7L2* and *NOS1AP* gene variants, may affect T2DM patients' responsiveness to hypoglycemic drugs [18–21].

As aforementioned, the biological function of the *MTNR1B* gene and the therapeutic effect of nateglinide are mainly focused on the regulation of insulin secretion and resistance. However, whether the efficacy of nateglinide gets affected by the *MTNR1B* gene variant remains unclear. Thus, in this study, *MTNR1B* rs10830963 gene was selected as a genetic marker and the effect of *MTNR1B* gene variant on the therapeutic efficacy of nateglinide in Chinese type 2 diabetes patients is determined.

## Materials and methods

### Study design and participants

This prospective case–control study included 200 unrelated T2DM patients (111 men and 89 women) and 200 healthy controls (99 men and 101 women) for analysis of *MTNR1B* rs10830963 gene variant. The T2DM patients and the healthy subjects were enrolled from the Department of Endocrinology and the Health Screening Center of the Affiliated Hospital of Xuzhou Medical College respectively. In the present study, the inclusion criteria for the control subjects were: (1) normal glucose tolerance as assessed by a standard 75 g OGTT (FPG < 6.1 mmol/L, PPG < 7.8 mmol/L); and (2) no family history of diabetes indicated in a standard questionnaire. Diagnosis of T2DM was carried out based on the 1999 World Health Organization (WHO) criteria for hyperglycemia under the following conditions: FPG > 7.0 mmol/L or postprandial plasma glucose (PPG) > 11.1 mmol/L. Exclusion criteria consisted of hypoglycemic drugs treatment, pregnancy or lactation, and the presence of serious diseases such as acute myocardial infarction, cerebral vascular accident, trauma, and kidney or liver diseases. All patients received a standard diabetes curriculum with a specific focus on diet, exercise and drug treatment compliance (Additional file 1).

A total of 60 newly diagnosed and unrelated T2DM patients (36 men and 24 women) with the same CYP2C9*1 and *SLCO1B1* 521TT genotypes were recruited for analysis of *MTNR1B* rs10830963 gene variant. They were subjected to detailed interviews and rigorous evaluations, including medication history. Patients who had not taken melatonin were included. Due to the close relationship between melatonin and *MTNR1B*, it is also necessary to exclude patients receiving this drug. All patients were asked to take 360 mg nateglinide per day (120 mg before each meal) orally for eight consecutive weeks. They were also advised of the same standard of diet control and exercise therapy. Inclusion criteria: (1) Newly diagnosed and unrelated T2DM patients, (2) with a body mass index (BMI) of 18.5–30 kg/m^2^. Exclusion criteria: (1) Have been treated with hypoglycemic drugs, (2) Those who had received agonists or inhibitors of CYP2C8, CYP2C9, CYP3A4 and SLCO1B1 treatment in recent 3 months, and (3) Patients who had received melatonin.

This study was registered in the Chinese Clinical Trial Register (No. ChiCTR13003536) and obtained approval from the ethics committee of the Affiliated Hospital of Xuzhou Medical College and followed the Helsinki Declaration II. Written informed consent was obtained from each participant before the study.

### Genotyping analysis

SiMax Genome DNA Kit (Sbsbio, Shanghai, China) was used to isolate the genomic DNA from the peripheral blood leucocytes. High resolution of melting curve (HRM) method was used to analyze the *MTNR1B* rs10830963 gene variant. Following primer pairs were used for the analyses: 5′-GAGGATTTGCTTGCTGAACA-3′ (forward) and 5′-CCCAGGCAGTTACTGGTTCT-3′ (reverse). The total HRM reaction system for detecting *MTNR1B* gene mutation was 20 μL, including 10 μL of HRM MasterMix buffer, 2.4 μL of Mg^2+^(25 mmol/L), 0.4 μL of each of the forward and reverse primers(10 mmol/L), and 5 μL of DNA(2 mg/L) and water was added to 20 μL. Cycle parameters: 95 °C for 10 min, 95 °C for 10 s, 65 °C for 15 s, 72 °C for 15 s, a total of 55 cycles. Melting: 95 °C 1 min, 40 °C 1 min, 70 °C 1 s, 95 °C 1 min. Cooling: 40 °C 30 s. Polymerase chain reaction-restriction fragment length gene variant (PCR–RFLP) was used for genotyping of *CYP2C9* gene variant and the four primer pairs used include forward primer: 5′-TGCACGAGGTCCAGAGATGC-3′, reverse primer: 5′-CTATGAATTTGGGGACTTCG-3′. Amplification refractory mutation system (ARMS) was used to detect the *SLCO1B1* T521C genotypes and the four primer pairs used include: forward primer: 5′-AAGTAGTTAAATTTGTAATAGAAATGC-3′, reverse primer: 5′-GTAGACAAAGGGAAAGTGATCATA-3′; forward primer for TT genotype: 5′-GGGTCATACATGTGGATATAAGT-3′, reverse primer for mutant variants: 5′-AAGCATATTACCCATGAACG-3′. 2% agarose gel electrophoresis was used to separate the obtained DNA fragments followed by ethidium bromide staining and visualization with UV transillumination.

### Clinical laboratory tests

Blood samples were collected from participants in fasting state (fasting for more than 8 h) and 2 h after breakfast respectively. 100 g of sugar-free steamed bread was provided for standard breakfast. Body parameters that included body height, body mass index (BMI), waist circumference, hip circumference, systolic blood pressure (SBP), and diastolic blood pressure (DBP) were measured before and at 8 weeks of treatment respectively. BMI was calculated as weight (kg)/height (m)^2^. Waist-to-Hip Ratio (WHR) was calculated as waistline (cm)/hipline (cm). Clinical indicators were also detected before and at 8 weeks after the administration of nateglinide. Roche Cobas8000 analyzer (Roche, Basel, Switzerland) was used to detect the plasma glucose, serum lipids triglycerides (TG), total cholesterol (TC), low-density cholesterol (LDL-c) and high-density cholesterol (HDL-c) with standard laboratory methods. Electro-chemiluminescence assay (Roche, Shanghai China) was used to measure insulin levels. High-performance liquid chromatography (HPLC) was used to determine the amounts of glycated hemoglobin (HbA_1c_). Homeostasis model assessment for insulin resistance (HOMA-IR) and islet β cell function (HOMA-β) was calculated using the formula: HOMA-IR = fasting insulin (mU/L) × fasting plasma glucose (mmol/L)/22.5 and HOMA-β = 20 × FINS (mU/L)/[FPG (mmol/L) − 3.5] respectively.

### Statistical analysis

Statistical analyses were performed with spss 18.0 software (SPSS, Chicago, IL, USA). The Hardy–Weinberg equilibrium, *allelic* frequencies in different groups, and categorical variables (counting data) were assessed using the Pearson chi-square test. All continuous variables were expressed as mean ± standard deviation (mean ± SD). The paired Student’s *t*-test was used to compare all the parameters between the two groups before and after nateglinide treatment. The two-sample *t* test or one-way ANOVA test were used for comparison between the two groups for the parameters of normal distribution. Parameters with abnormal distribution were analyzed by the Kruskal–Wallis test. Statistical power calculations were performed using a power calculator software PASS (www.ncss.com). A value of *P* < 0.05 was considered statistically significant.

## Results

### Allelic frequency analysis

A total of 200 T2DM patients (111 men and 89 women) and 200 healthy subjects (99 men and 101 women) were genotyped for *MTNR1B* rs10830963 gene variant. The genotype distribution in each group was consistent with the Hardy–Weinberg equilibrium (*P* > 0.05). The allele frequencies of the *MTNR1B* rs10830963 gene variant in T2DM patients and healthy subjects are given in Table 1. The frequency of the *MTNR1B* rs10830963 G *allele* was higher in T2DM patients when compared to the healthy subjects (42.50% vs 34.50%, *P* < 0.05).

**Table 1:** Comparison of genotype and frequencies of *MTNR1B* rs10830963 polymorphism between T2DM patients and healthy subjects

Genotypes	Healthy subjects (n = 200)	T2DM patients (n = 200)	*P* value
MTNR1B rs10830963			
CC	82(41.00%)	70(35.00%)	
CG	98(49.00%)	90(45.00%)	
GG	20(10.00%)	40(20.00%)	0.019^Δ^*
*Alleles*			
C	262(65.50%)	230(57.50%)	
G	138(34.50%)	170(42.50%)	0.020^Δ^*

The *allelic* frequencies are indicated in absolute values (percentage). ^Δ^*P* values are determined by the Pearson chi-square test. *P < 0.05

### Assessment of baseline parameters with different MTNR1B rs10830963 genotypes in T2DM patients

**Fig. 1:**
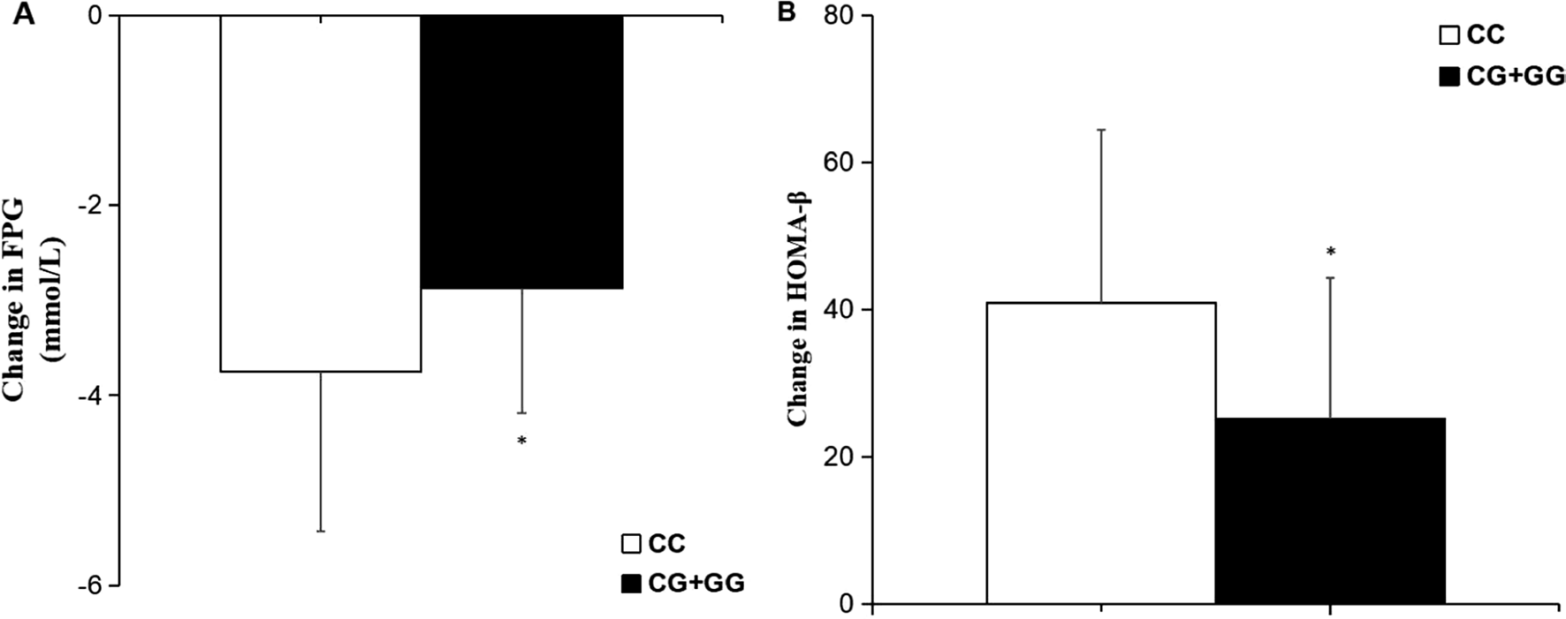
Baseline levels of FPG in T2DM patients with different MTNR1B rs10830963 genotypes. Data are expressed as (mean ± SD). *P < 0.05 compared with CC genotype group

The baseline clinical characteristics of T2DM patients with different *MTNR1B* rs10830963 genotypes were analyzed in Table 2. No association was observed between *MTNR1B* rs10830963 gene variant and sex, age, BMI, WHR, PPG, fasting serum insulin (FINS), postprandial serum insulin (PINS), HOMA-IR, HbA1c, TG, TC, HDL-c, and LDL-c. However, FPG, (9.61 ± 2.01 mmol/L for CC genotype, 9.91 ± 2.79 mmol/L for CG and 10.82 ± 1.79 mmol/L for GG, respectively; *P* < 0.05, Fig. 1) showed significant differences.

**Table 2:** The baseline characteristics in T2DM patients with various *MTNR1B* rs10830963 genotypes before treatment with nateglinide (n = 200)

Parameters	MTNR1B rs10830963 genotype			*P* value
	CC	CG	GG	
N (men/women)	70(40/30)	90(48/42)	40(23/17)	0.850^Δ^
Age (years)	47.81 ± 10.82	48.01 ± 12.04	47.09 ± 13.92	0.921
BMI (kg/m^2^)	26.41 ± 3.24	25.43 ± 3.31	26.59 ± 4.11	0.104
WHR	0.92 ± 0.06	0.91 ± 0.06	0.92 ± 0.08	0.552^#^
FPG (mmol/L)	9.61 ± 2.01	9.91 ± 2.79	10.82 ± 1.79	0.034*
PPG (mmol/L)	15.36 ± 2.46	14.21 ± 4.39	14.69 ± 5.71	0.224
FINS (mU/L)	8.56 ± 5.39	7.37 ± 6.99	7.53 ± 6.39	0.477^#^
PINS (mU/L)	30.01 ± 17.10	28.11 ± 20.51	33.51 ± 17.49	0.320
HOMA-IR	3.27 ± 1.30	3.09 ± 3.21	4.01 ± 2.47	0.160
HbA1c (%)	9.71 ± 1.97	9.11 ± 2.62	9.95 ± 2.04	0.098
TG (mmol/L)	2.25 ± 1.38	2.31 ± 2.13	1.97 ± 1.52	0.596
TC (mmol/L)	5.21 ± 1.29	4.99 ± 1.31	5.49 ± 1.51	0.143
HDL-c (mmol/L)	1.28 ± 0.95	1.33 ± 0.59	1.41 ± 0.29	0.645
LDL-c (mmol/L)	3.45 ± 0.68	3.61 ± 1.19	3.80 ± 0.93	0.199^#^

*BMI* body mass index, *WHR* waist to hip ratio, *FPG* fasting plasma glucose, *PPG* postprandial plasma glucose, *FINS* fasting serum insulin, *PINS* postprandial serum insulin, *HOMA-IR* homeostasis model assessment for insulin resistance, *HbA*_*1c*_ hemoglobin A_1c_, *TG* triglyceride, *TC* total cholesterol, *HDL-c* high-density lipoprotein-cholesterol, *LDL-c* low-density lipoprotein-cholesterol

Data are given as (mean ± SD). *P* values represent statistical differences among the three different genotypes by the one-way ANOVA. ^Δ^*P* values are determined by the Pearson chi-square test. ^#^*P* values are determined by the Kruskal–Wallis test. *P < 0.05

### Effects of MTNR1B rs10830963 gene variant on the efficacy of nateglinide in T2DM patients

T2DM patients (n = 60) with different *MTNR1B* rs10830963 but the same *SLCO1B1* 521TT and CYP2C9*1 genotypes were randomly selected to participate in our study to avoid the potential impacts of *SLCO1B1* and *CYP2C9* genetic polymorphisms. It was observed that these patients responded to nateglinide therapy. After 8 weeks of treatment, they showed a remarkable decline in the level of FPG, PPG, HbA_1c_ and TC (all *P* < 0.05), but significant increase in the levels of FINS, PINS and HOMA-β (all *P* < 0.05). The comparison with the pretreatment values was tabulated in Table 3.

**Table 3:** Clinical characteristics of T2DM patients before and after nateglinide treatment (n = 60)

Parameters	Before treatment n = 60	After treatment n = 60	*P* values
BMI (kg/m^2^)	25.41 ± 3.54	25.33 ± 3.11	0.896
WHR	0.92 ± 0.06	0.91 ± 0.06	0.363
FPG (mmol/L)	10.41 ± 1.23	7.25 ± 1.21	0.000*
PPG (mmol/L)	14.56 ± 2.76	9.38 ± 2.61	0.000*
FINS (mU/L)	7.56 ± 4.39	9.51 ± 3.46	0.008*
PINS (mU/L)	30.31 ± 17.10	47.21 ± 15.16	0.000*
HOMA-IR	3.57 ± 1.30	2.72 ± 1.41	0.001*
HOMA-β	25.73 ± 14.5	60.32 ± 21.15	0.000*
HbA1c (%)	9.31 ± 1.87	7.89 ± 0.81	0.000*
TG (mmol/L)	2.25 ± 1.38	1.86 ± 1.23	0.105
TC (mmol/L)	5.21 ± 1.29	4.31 ± 1.09	0.000*
HDL-c (mmol/L)	1.38 ± 0.75	1.45 ± 0.59	0.571
LDL-c (mmol/L)	3.85 ± 1.78	3.42 ± 1.07	0.111

*BMI* body mass index, *WHR* waist to hip ratio, *FPG* fasting plasma glucose, *PPG* postprandial plasma glucose, *FINS* fasting serum insulin, *PINS* postprandial serum insulin, *HOMA-IR* homeostasis model assessment for insulin resistance, *HOMA-β* Homeostasis model assessment for islet β cell function, *HbA1c* hemoglobin A1c, *TG* triglyceride, *TC* total cholesterol, *HDL-c* high-density lipoprotein-cholesterol, *LDL-c* low-density lipoprotein-cholesterol

Data are expressed as (mean ± SD). *P* values are determined by the paired Student’s *t* test. *P < 0.05

**Fig. 2:**
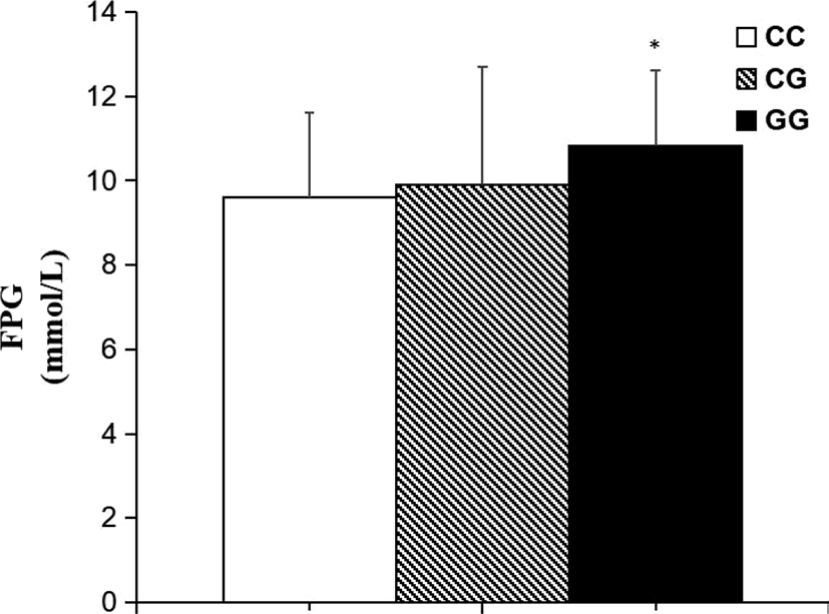
Comparisons of differential values (pre-administration levels subtracted from the post-administration levels) of FPG (**A**) and HOMA-β (**B**) between different MTNR1B rs10830963 genotypes in T2DM patients before and after treatment of nateglinide. Data are expressed as (mean ± SD). *P < 0.05 compared with CC genotype group respectively

Since the GG genotype frequency was lower in the selected population, we combined the CG genotype (26 cases) and the GG genotype (8 cases) for analysis and compared with the CC genotype (26 cases). After nateglinide treatment, the FPG value of the patients with genotypes CG and GG was higher, when compared with the carriers of genotype CC. PINS and HOMA-β values were lower, when compared with the CC genotype carriers (*P* < 0.05). T2DM patients with genotype CC at *MTNR1B* rs10830963 had a significant decrease in FPG (mmol/L) when compared with the genotypes CG and GG (− 3.75 ± 1.68 vs − 2.87 ± 1.32; *P* < 0.05) respectively. In addition, the carriers of genotype CC at MTNR1B rs10830963 had higher differential values of HOMA-β, when compared with the genotypes CG and GG (40.87 ± 23.52 vs 25.13 ± 19.21; *P* < 0.05) respectively (Table 4, Fig. 2).

**Table 4:** Effects of different *MTNR1B* rs10830963 genotypes in T2DM patients on clinical traits determined before and after nateglinide treatment

Parameters	CC(n = 26)	CG(n = 26) + GG(n = 8)	*P* value
N (male/female)	26 (16/10)	34(20/14)	0.902^Δ^
FPG (mmol/L)			
Before	10.73 ± 2.05	10.81 ± 1.92	0.877
After	6.98 ± 1.35	7.94 ± 1.23	0.006*
DV	− 3.75 ± 1.68	− 2.87 ± 1.32	0.027*
PPG (mmol/L)			
Before	14.51 ± 4.31	13.04 ± 4.72	0.220
After	9.34 ± 3.24	9.27 ± 3.49	0.937
DV	− 5.17 ± 4.08	− 3.77 ± 3.19	0.141^#^
FINS (mU/L)			
Before	8.82 ± 6.81	7.80 ± 4.20	0.478
After	10.18 ± 6.60	9.32 ± 3.64	0.522
DV	1.36 ± 4.10	1.84 ± 3.80	0.641
PINS (mU/L)			
Before	31.42 ± 20.47	27.47 ± 16.43	0.410
After	48.41 ± 19.78	37.13 ± 20.87	0.038*
DV	16.99 ± 17.82	9.66 ± 19.37	0.138
HOMA-IR			
Before	4.48 ± 2.88	3.24 ± 1.73	0.066
After	3.39 ± 2.12	2.43 ± 1.18	0.072
DV	− 0.89 ± 1.93	− 0.79 ± 1.31	0.704
HOMA-β			
Before	27.75 ± 16.03	24.08 ± 17.32	0.405
After	68.62 ± 45.21	49.21 ± 24.36	0.037*
DV	40.87 ± 23.52	25.13 ± 19.21	0.006*
HbA1c (%)			
Before	9.79 ± 1.86	9.15 ± 2.26	0.246^#^
After	7.00 ± 0.84	7.01 ± 1.05	0.968
DV	− 2.79 ± 1.53	− 2.14 ± 1.74	0.137
TG (mmol/L)			
Before	2.46 ± 1.96	2.31 ± 1.90	0.766
After	2.21 ± 1.91	1.79 ± 1.23	0.306
DV	− 0.25 ± 1.95	− 0.52 ± 1.58	0.096^#^
TC (mmol/L)			
Before	5.27 ± 1.69	5.22 ± 1.15	0.892^#^
After	4.84 ± 1.12	4.89 ± 1.37	0.880^#^
DV	− 0.43 ± 1.53	− 0.33 ± 1.09	0.769^#^
HDL-c (mmol/L)			
Before	1.42 ± 0.51	1.47 ± 0.47	0.695^#^
After	1.35 ± 0.55	1.43 ± 0.51	0.563^#^
DV	− 0.07 ± 0.68	− 0.04 ± 0.46	0.839
LDL-c (mmol/L)			
Before	3.28 ± 1.19	3.18 ± 1.05	0.731^#^
After	3.19 ± 1.20	3.11 ± 1.07	0.786
DV	− 0.09 ± 1.26	− 0.07 ± 1.08	0.948

*BMI* body mass index, *WHR* waist to hip ratio, *FPG* fasting plasma glucose, *PPG* postprandial plasma glucose, *FINS* fasting serum insulin, *PINS* postprandial serum insulin, *HOMA-IR* homeostasis model assessment for insulin resistance, *HOMA-β* Homeostasis model assessment for islet β cell function, *HbA1c* hemoglobin A1c, *TG* triglyceride, *TC* total cholesterol, *HDL-c* high-density lipoprotein-cholesterol, *LDL-c* low-density lipoprotein-cholesterol, *DV* differential values (post-administration minus pre-administration)

Data are given as (mean ± SD). ^Δ^*P* values are determined by the Pearson chi-square test. ^#^*P* values are determined by the Kruskal–Wallis test. *P < 0.05

## Discussion

In this study, the gene variant of *MTNR1B* rsl0830963 potentially influenced the efficacy of nateglinide in Chinese patients with T2DM. We observed this in T2DM patients with G allele of *MTNR1B* rs10830963 decreased the efficacy of nateglinide. We also found that the risk G *allelic* frequency of *MTNR1B* rsl0830963 was significantly higher in T2DM patients than healthy subjects (*P* < 0.05). Consequently, *MTNR1B* gene represents a susceptibility target for T2DM and affects the response to nateglinide.

A significant basis for clinical individualized drug delivery is provided by a relatively new subject called pharmacogenomics. Presently, the pharmacogenomics researches of oral hypoglycemic drugs mainly focus on the study of classic sulfonylurea oral hypoglycemic agents, thiazolidinedione insulin sensitizers and biguanide hypoglycemic drugs. There are few reports on the pharmacogenomics of the novel insulin secretagogues of glinide, but no evidence related to *MTNR1B* gene is found.

Studies conducted with the Chinese Han population have confirmed that single nucleotide gene variants (SNPs) of *MTNR1B* are associated with T2DM susceptibility [22, 23]. Among the SNP sites involved in *MTNR1B*, the rs10830963 locus is a functional polymorphic locus and is closely related to glucose metabolism and islet β cell function. Further, it is highly correlated with the pathogenesis of T2DM. The variation of the *MTNR1B* gene is consistent with the FPG level; rs10830963 is the most relevant, and each G allele increases the FPG level by 0.07 mmol/L. The homeostasis model assessment (HOMA-β) analysis showed impaired beta-cell function [15]. In this study, we found that the risk G *allelic* frequency of *MTNR1B* rsl0830963 was significantly higher in T2DM patients, when compared to the healthy subjects (*P* < 0.05) and remains consistent with previous studies [15, 16]. The study had an estimated 80–92% power (for α = 0.05) to detect such a difference in allelic frequencies and genotypes distribution. Also, it is found that patients with T2DM carrying the GG genotype had higher FPG levels when compared with the CC and CG genotypes. The difference being statistically significant, suggest that the G allele has an association with the elevated FPG levels.

The pineal gland releases the circulating hormone melatonin (MLT) and its action is mediated by melatonin receptor 1 and 2 (MT1, MT2) respectively. MT2 is encoded by the *MTNR1B* gene and is expressed in the islet beta cells of both animals and human beings [13]. Multiple GWAS studies conducted in European populations found that *MTNR1B* rs10830963 is associated with FPG, insulin secretion, and T2DM susceptibility [14, 15]. Subsequently, it was also found that *MTNR1B* rs10830963 has an association with FPG and islet β-cell function in Chinese Han population [16, 17]. However, the molecular mechanism by which the *MTNR1B* gene variant increases T2DM susceptibility remains unclear. Studies have reported that after MLT activates MT2, MT2 gets coupled with the inhibitory G protein, mediating cAMP and cGMP signal transduction pathways and inhibits insulin release from islet beta cells [22, 23]. In addition to MLT in *MT2* knockout mice, islet β cells release insulin increases [24] and therefore *MTNR1B* gene variant increased T2DM susceptibility relating to its influence on insulin secretion.

Next to repaglinide, nateglinide is the new non-sulfonylurea oral hypoglycemic agent. It is more commonly used in clinical practices, but the difference in efficacy and adverse reactions is significant. The main mechanism of action of nateglinide is to close the ATP-dependent K^+^ channel on the islet β-cell membrane to cause the depolarization of the cell membrane and open the Ca^2+^ channel to lead to Ca^2+^ influx and thus promote insulin secretion [25]. Therefore, the *MTNR1B* gene variant plays a role in the hypoglycemic effect of nateglinide.

The purpose of this study was to analyze the effect of *MTNR1B* rs10830963 gene variant on the efficacy of nateglinide in treating the newly diagnosed type 2 diabetes patients. Previous studies have reported that *CYP2C9* and *SLCO1B1* gene variants may affect the pharmacokinetics of nateglinide [26–29]. Hence we decided to retain the same patients with the CYP2C9*1 and *SLCO1B1* 521TT genotypes as subjects to rule out interference. After 8 consecutive weeks of nateglinide monotherapy, patients with FPG, PPG, FINS, PINS, HOMA-IR, HOMA-β, HbA1c, and TC showed significant improvement. This suggested that nateglinide has a good therapeutic effect on patients with type 2 diabetes. There are literatures reporting the nateglinide effect on improving insulin resistance [10, 11]. Our research results were found to be consistent with the literature results. But, there was no evidence to find the relationship between *MTNR1B* rs10830963 gene variant and nateglinide efficacy. Therefore, in our study, we compared the difference between the clinical indicators before and after nateglinide treatment. The decrease of FPG and the increase of HOMA-β in *MTNR1B* rs10830963 risk gene G carriers were lower when compared with the CC genotype patients (*P* < 0.05). These results indicated that the risk gene G carriers had a worse response to nateglinide when compared with the CC genotype patients. Also, the clinical treatment showed that the GG genotype patient had poor nateglinide treatment. Prokopenko et al [15] reported that calculation of islet beta-cell function using the homeostasis model showed that, *MTNR1B* rs10830963 risk gene G carriers had lower islet function. Lyssenko et al. [14] found “in” GG homozygotes, oral or intravenous glucose stimulation early-phase insulin release was impaired. Previous reports results were consistent with the results of this study. After nateglinide treatment, risk gene G may further reduce the efficacy of nateglinide by affecting FPG and HOMA-β. The exact mechanism by which the *MTNR1B* gene variant affects the efficacy of nateglinide requires further investigation.

However, this study does have some shortcomings as the sample size is not large enough, and the frequency of *MTNR1B* rs10830963 GG genotype is low. Therefore, this study might miss some meaningful results. Hence, we recommend further detailed study with expanded sample size. Glinide drugs are mealtime blood glucose regulators and are characterized by rapid but short-acting insulin secretion with weak hypoglycemic effect and good safety. Therefore, this study neither focused on the clinical adverse events during nateglinide monotherapy nor did it receive reports of adverse events in the subjects. T2DM is a multi-gene metabolic disease and in this study we found that the *MTNR1B* gene variant has a certain effect on the efficacy of nateglinide. But the individual difference in the efficacy of hypoglycemic drugs is caused by the accumulation of multiple gene variants as well as the changes in the environmental factors and lifestyles. The results of a single genetic polymorphism study could not fully explain the individual differences in drug efficacy. Patients received only an 8-week course of nateglinide therapy, and optimal reduction in HbA1c levels occurs after 12 weeks of administration [30]. Therefore, in order to apply this research results to clinical practice, it requires collaboration among researchers from different regions.

In summary, the results of this study suggest that the *MTNR1B* rs10830963 gene variant is associated with the efficacy of nateglinide in the treatment of type 2 diabetes, and also has a certain role in promoting clinical individualized drug delivery. Finally, the statistical power of our study may be sufficient had we collected a relatively larger sample size. We recommend exploration with more extensive and comprehensive clinical research along with an in-depth mechanism to confirm its relevance.

## Conclusion

In conclusion, we report for the first time that *MTNR1B* rs10830963 gene variant might influence the incidence of T2DM among the Chinese Han population and the efficacy of nateglinide monotherapy. The CC homozygotes had a better effect than G *allele* carriers. Genetic, environmental and disease factors may be the important reasons for individual differences in drug efficacy. Genetic pharmacology mainly studies the influence of genetic factors on individual differences in drug efficacy, which provides the basis for further mechanism exploration and clinical individualized drug administration. With in-depth studies about genetic contributors to diabetes treatment response, it is more likely that the management of diabetes disease will be at the forefront of translating exploratory research into clinical practice in some situations. Indeed, further pharmacogenetic and functional investigations are required to determine the effect of *MTNR1B* variants on nateglinide therapeutic efficacy and to lay the foundation for patient-tailored therapy.

## Supplementary Information

**Additional file 1**. Dietarycontrol compliance assessment form.

## Data Availability

The datasets generated and analyzed during the current study are available in the link “https://submit.ncbi.nlm.nih.gov/subs/variation_file/SUB9598166/overview”. The accession number is SUB9598166.

## Abbreviations

HRM: High resolution of melting curve
T2DM: Type 2 diabetes mellitus
FPG: Fasting plasma glucose
GWAS: Genome-wide association studies
WHO: World Health Organization
BMI: Body mass index
PCR-RFLP: Polymerase chain reaction-restriction fragment length polymorphism
ARMS: Amplification refractory mutation system
SBP: Systolic blood pressure
DBP: Diastolic blood pressure
WHR: Waist-to-hip ratio
TG: Triglycerides
TC: Total cholesterol
LDL-c: Low-density cholesterol
HDL-c: High-density cholesterol
HPLC: High-performance liquid chromatography
HbA1c: Glycated hemoglobin
HOMA-IR: Homeostasis model assessment for insulin resistance
HOMA-β: Homeostasis model assessment for islet β cell function
FINS: Fasting serum insulin
PINS: Postprandial serum insulin
