# Supplementary material for: Evaluation of the effect of MTNR1B rs10830963 gene variant on the therapeutic efficacy of nateglinide in treating type 2 diabetes among Chinese Han patients
Source: BMC Med Genomics. 2021 Jun 12;14:156. doi: 10.1186/s12920-021-01004-y (PMC8196487; doi:10.1186/s12920-021-01004-y)
Supplement: Supplementary file 1 — Additional file 1. Dietarycontrol compliance assessment form. [file 12920_2021_1004_MOESM1_ESM.doc]

**Dietary control compliance assessment form**

Jin‑Fang Song1†, Jie Zhang2†, Ming‑Zhu Zhang3, Jiang Ni1, Tao Wang4, Yi‑Qing Zhao1* and Naveed Ullah Khan5*

*Correspondence: 13813454165@126.com; naveedkhan1676@hotmail.com

^1^ Department of Pharmacy, Afliated Hospital of Jiangnan University

(Wuxi Third People’s Hospital), No. 585, Xingyuan North Road,

Wuxi 214000, China

^5^ Department of Pharmaceutics, College of Pharmaceutical Sciences,

Soochow University, Suzhou, Jiangsu, China

Guidance: Please select the appropriate option from "fully compliant", "basically compliant", "basically non compliant" and "completely noncompliant" according to your situation, and mark "√" in the corresponding part.

| Question | Answer | | | |
| --- | --- | --- | --- | --- |
|  | fully compliant | basically compliant | basically noncompliant | completely noncompliant |
| 1 I don't plan to eat according to the rules of diabetes | 0 | 1 | 2 | 3 |
| 2 I was educated in formal dietotherapy | 3 | 2 | 1 | 0 |
| 3 I think the diabetes diet is too cumbersome, so I can't carry it out well | 0 | 1 | 2 | 3 |
| 4 I feel like I'm not eating the same way as the rest of my family, and I'm causing them a lot of trouble |  |  |  |  |
| 5 It bothers me that I can't eat like a normal person |  |  |  |  |
| 6 I think it's a lot of trouble for me to eat according to the food exchange law |  |  |  |  |
| 7 It's too troublesome to choose the time of diet, the type of food and the amount of food according to the diet habit of diabetes mellitus |  |  |  |  |
| 8 I can't help myself to eat more |  |  |  |  |
| 9 I had to give up my favorite food |  |  |  |  |
| 10 I am often too hungry to bear |  |  |  |  |
| 11 I can strictly control my diet |  |  |  |  |
| 12 I can still control my diet when I eat with people I don't know |  |  |  |  |
| 13 I can still control my diet when I go out to familiar places (friends, relatives) |  |  |  |  |
| 14 I can still control my diet during holidays, birthdays or outings |  |  |  |  |
| 15 As the intensity of work changes, so does diet |  |  |  |  |
| 16 As the weight changes, so does the diet |  |  |  |  |
| 17 What I eat is often not to my taste |  |  |  |  |
| 18 My family supports my diabetes diet |  |  |  |  |
| 19 When I'm in a bad mood, I drink and smoke |  |  |  |  |
| 20 I think my diet therapy is very successful |  |  |  |  |

Note: According to the different questions, the selections will be scored 0-3 points. The total score is 60 points, and 48 points is considered that the compliance of the subject reaches the standard.

**饮食控制依从情况评估表**

指导语：请根据您的情况，从“完全符合”、“基本符合”、“基本不符合”、“完全不符合”中选择合适您自己的选项，在相应的部分打“√”。

| 题目 | 回答 | | | |
| --- | --- | --- | --- | --- |
|  | 完全符合 | 基本符合 | 基本不符合 | 完全不符合 |
| 1 我没有打算按照糖尿病的规定饮食进餐 | 0 | 1 | 2 | 3 |
| 2 我接受过正规饮食疗法教育 | 3 | 2 | 1 | 0 |
| 3 我觉得糖尿病饮食方案太繁琐，所以我不能很好地执行 | 0 | 1 | 2 | 3 |
| 4 我觉得自己与家里其他人的饮食方式不同，给他们带来了许多麻烦 |  |  |  |  |
| 5 我不能像正常人一样吃东西，这让我感到烦恼 |  |  |  |  |
| 6 我觉得按照食品交换法进餐给我带来很多麻烦 |  |  |  |  |
| 7 按照糖尿病饮食习惯选择饮食时间、食物的种类及饮食量太麻烦 |  |  |  |  |
| 8 我总是控制不住自己多吃东西 |  |  |  |  |
| 9 我不得不放弃自己喜欢的食物 |  |  |  |  |
| 10 我经常饿得难以忍受 |  |  |  |  |
| 11 我平时能够严格控制饮食 |  |  |  |  |
| 12 与不熟识的人一起用餐时我仍能控制饮食 |  |  |  |  |
| 13 出门到熟识的地方（朋友、亲戚）我仍能控制饮食 |  |  |  |  |
| 14 在节假日、生日或出外应酬时，我仍能控制饮食 |  |  |  |  |
| 15 当工作强度改变时，饮食也随之调整 |  |  |  |  |
| 16 当体重改变时，饮食也随之调整 |  |  |  |  |
| 17 我所吃的东西经常不合胃口 |  |  |  |  |
| 18 家里人支持我采用糖尿病饮食疗法 |  |  |  |  |
| 19 当心情不好时，我通过喝酒、吸烟来排解 |  |  |  |  |
| 20 我认为自己的饮食疗法执行的很成功 |  |  |  |  |

注：根据不同问题，受访者选择完全符合、基本符合、基本不符合、完全不符合，研究者将按0-3分评分。总分60分，≥48为依从性达标。

**Exercise therapy compliance assessment questionnaire**

During the follow-up, all patients were investigated and evaluated for compliance of exercise therapy

1. Do you exercise more than 5 times a week?

2. Does each exercise last more than 30 minutes?

3. Whether to choose the exercise time according to the specific situation?

4. Is the exercise intensity appropriate?

If the answer is "yes" or "no", the compliance of exercise therapy will be assessed as up to standard. If only one of the answers is "no", it will be assessed as substandard compliance of exercise therapy.

**运动疗法依从性评估问卷**

回访时对所有病人进行运动疗法依从性达标调查评价, 内容包括:

1. 每周运动次数, 是否在5次以上？
2. 每次运动时间是否超过30min？
3. 是否根据具体情况选择运动时间？
4. 运动强度是否适宜?

每项以是与否作答, 若回答均为是，则评定为运动疗法依从性达标，只要有1项回答为否，则评定为运动疗法依从性不达标。
